# *Plasmodium* female gamete surface HSP90 is a key determinant for fertilization

**DOI:** 10.1128/mbio.03142-23

**Published:** 2023-12-22

**Authors:** Sung-Jae Cha, Joel Vega-Rodriguez, Dingyin Tao, Heather M. Kudyba, Kelly Hanner, Marcelo Jacobs-Lorena

**Affiliations:** 1 Department of Medical Sciences, Mercer University School of Medicine, Macon, Georgia, USA; 2 Laboratory of Malaria and Vector Research, National Institute of Allergy and Infectious Diseases, National Institutes of Health, Rockville, Maryland, USA; 3 Department of Molecular Microbiology and Immunology and Malaria Research Institute, Johns Hopkins Bloomberg School of Public Health, Baltimore, Maryland, USA; Washington University in St. Louis School of Medicine, Saint Louis, Missouri, USA

**Keywords:** phage display, male binding peptide, *Plasmodium *fertilization, transmission blocking, HSP90

## Abstract

*Plasmodium* fertilization, an essential step for the development of the malaria parasite in the mosquito, is a prime target for blocking pathogen transmission. Using phage peptide display screening, we identified MG1, a peptide that binds to male gametes and inhibits fertilization, presumably by competing with a female gamete ligand. Anti-MG1 antibodies bind to the female gamete surface and, by doing so, also inhibit fertilization. We determined that this antibody recognizes HSP90 on the surface of *Plasmodium* female gametes. Our findings establish *Plasmodium* HSP90 as a prime target for the development of a transmission-blocking vaccine.

**IMPORTANCE:**

Malaria kills over half a million people every year and this number has not decreased in recent years. The development of new tools to combat this disease is urgently needed. In this article, we report the identification of a key molecule—HSP90—on the surface of the parasite’s female gamete that is required for fertilization to occur and for the completion of the parasite cycle in the mosquito. HSP90 is a promising candidate for the development of a transmission-blocking vaccine.

## INTRODUCTION

Malaria is caused by parasites of the genus *Plasmodium* and is the world’s most devastating parasitic disease (1). Transmission of malaria parasites by anopheline mosquitoes is absolutely dependent on successful completion of the pathogen’s sexual reproduction in the mosquito midgut. When a mosquito ingests an infected blood meal, the *Plasmodium* sexual forms, known as gametocytes, are exposed to mosquito midgut signals that induce activation of gametocytes into gametes (2). Within 15 min, activated gametocytes egress from red blood cells (RBCs) as gametes. Male gametes undergo a morphological and physiological transformation known as exflagellation during which DNA undergoes three rapid rounds of replication, leading to the formation of eight microgametes (3). Microgametes detach from the exflagellation center and actively move in search of female gametes in the mosquito blood bolus to attach, fuse, and complete fertilization. *Plasmodium* gamete fertilization is considered a promising target for transmission-blocking vaccines (TBVs).

TBVs consist of antibodies that bind to parasite antigens to block development in the mosquito midgut. Three promising TBV antigen candidates, P47, P48/45, and P230, belong to the 6-cysteine protein family that are expressed on the surface of gametes (4–6). Disruption of P48/45 or P230 produces infertile male gametes and disruption of P47 results in infertile female *Plasmodium berghei* gametes. However, complete inhibition of fertilization is not achieved, even after the simultaneous disruption of P47 and P48/45 (7). Hapless 2 is another TBV candidate essential for gamete membrane fusion (8). In addition, the P25 and P28 major surface proteins that are expressed after zygote formation have also been investigated as TBV antigen candidates. The rationale for TBVs is that transmission-blocking antibodies will interfere with molecular interactions essential for parasite development in the mosquito. However, to date, no molecular interacting (receptor-ligand) partners have been identified for any of the above-mentioned TBV candidates or any other gamete surface protein required for fertilization. This much-needed information would further our understanding of the biology of malaria fertilization as well as improve the current efforts of developing a successful TBV.

We have previously used a phage display library approach to identify receptor-ligand interactions for parasite invasion of the mosquito midgut and salivary glands, and of the mammalian liver (9–13). Also, we reported on the identification of the female gamete-binding peptide 1 (FG1) which binds to *Plasmodium* female gametes and strongly inhibits fertilization and parasite development in the mosquito vector (14). Here, we report on the identification of heat shock protein 90 (HSP90), a female gamete surface protein required for fertilization and a TBV candidate.

## RESULTS

### Identification of the male gamete-binding peptide MG1 that inhibits oocyst formation

We performed a phage display library screen for peptides with high affinity to the surface of male gametes (Fig. S1A). Gametocytes of the transgenic *P. berghei* 820cl1m1cl1 line that produces female gametocytes expressing Red Fluorescent Protein (RFP) (red) and male gametocytes expressing Green Fluorescent Protein (GFP) (green) (14) were incubated in *P. berghei* ookinete culture medium to induce gamete formation. To screen peptides that bind to activated male gametes, aphidicolin, a DNA synthesis inhibitor, was added to inhibit further steps including exflagellation and fertilization. GFP-expressing male gametes were isolated by cell sorting (Fig. S1B) and incubated with a phage library. The phages in this library display 12-amino acid peptides fused to the pVIII coat protein. The library has a complexity of 1.5 × 10^9^ different peptides with random amino acid compositions except for the cysteines at positions 2 and 11 that make a disulfide bond generating an eight-amino acid loop (15). After three rounds of selection, 64% of the recovered phages displayed the same peptide, designated male gamete peptide 1 (MG1). This peptide is exceptional in that one of the two cysteines has mutated to a phenylalanine (Fig. S1C). Passive administration feeding assays, in which mosquitoes are fed on a *P. berghei*-infected mouse before and after intravenous phage injection, showed that phages expressing the MG1 peptide inhibit oocyst formation by 69%, whereas wild-type control phage showed a baseline 35% inhibition (Fig. S2A, B and S3).

### The MG1 peptide selectively binds to the male gamete surface

**Fig 1:**
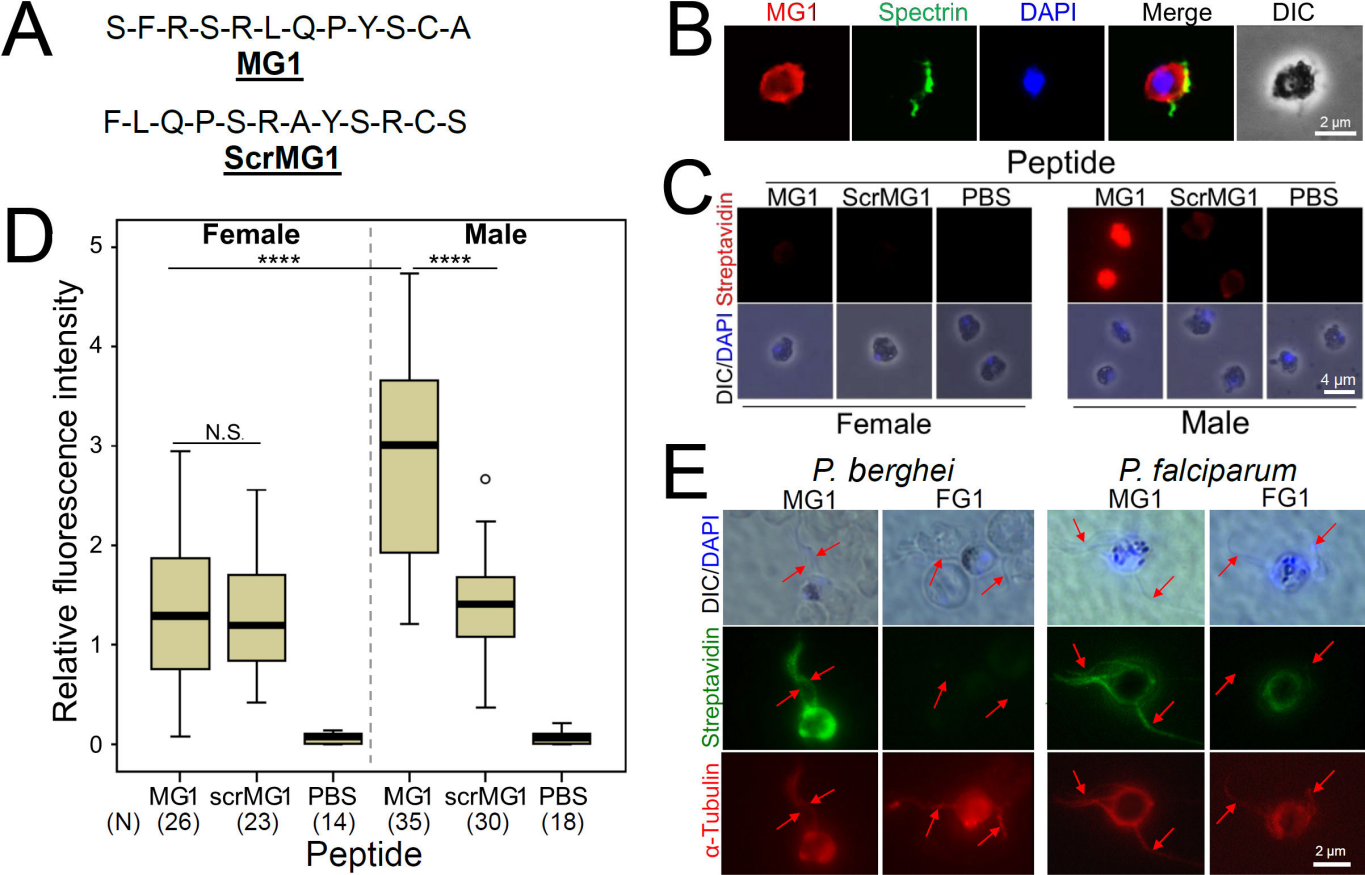
The synthetic MG1 peptide specifically binds to *Plasmodium* male gametes. (**A**) Amino acid sequence of the MG1 peptide and of the scrambled version (ScrMG1). The amino acid composition of MG1 and ScrMG1 is the same. (**B**) Biotinylated-MG1 binding (red) to an activated *P. berghei* male gamete. A cortical cytoskeleton fragment of the former host RBC stained by an anti-spectrin antibody (green) shows gamete egress. (**C**) Representative images showing biotinylated-MG1 or -ScrMG1 binding to sorted *P. berghei* female and male gametes. Peptide binding to gametes was visualized with Texas-red-conjugated streptavidin. The original RFP and GFP fluorescence are lost after fixation. (**D**) Relative fluorescence intensity (RFI) due to MG1 or ScrMG1 peptide binding to gametes as exemplified in (**C**) was quantified from fluorescent images using the Image J software. The vertical bar in the box plot shows the maximum and the minimum RFI, and the thick horizontal lines within the boxes show the median RFI. *P*-values (****, <0.0001) were calculated with the one-way Analysis of Variance-Honestly Significant Difference (ANOVA-HSD) test. The numbers in parenthesis denote total number (N) of gametes analyzed. N.S., not significant. (**E**) Binding of biotinylated MG1 peptide to *P. berghei* and *P. falciparum* microgametes. Fixed microgametes were labeled with an Allophycocyanin (APC)-conjugated anti-alpha-tubulin antibody and MG1 or the female-specific FG1 peptide as a negative control. Arrows point to microgamete flagella. Peptide binding was visualized with Alexa488-conjugated streptavidin. DIC, differential interference contrast microscopy; DAPI, nuclear stain (blue).

To exclude steric hindrance by the ~1 μm-long phage particles, further experiments were performed with synthetic biotinylated MG1 and ScrMG1, a control peptide with the same amino acid composition but with scrambled order (Fig. 1A). Gametocyte activation was performed in the presence of aphidicolin to prevent exflagellation and fertilization. Gamete egression from RBCs was confirmed by immunostaining of spectrin, a component of the RBC membrane cytoskeleton (Fig. 1B). The binding specificity of biotinylated MG1 and ScrMG1 peptides to *P. berghei* gametes was determined by quantifying the fluorescence intensity after peptide labeling with streptavidin-conjugated to Texas-red. The MG1 peptide binds specifically to the surface of non-exflagellated male gametes in the presence of aphidicolin, whereas binding to female gametes was significantly weaker (Fig. 1C and D: *P* < 0.0001). ScrMG1 showed reduced binding affinity to male gametes, confirming the specificity of the MG1 interaction (Fig. 1C and D). Furthermore, MG1 also binds to the surface of *P. berghei* and *Plasmodium falciparum* microgametes (after exflagellation), as shown by labeling with Alex488-conjugated streptavidin (Fig. 1E). The female-specific peptide FG1, previously shown to not bind to *P. berghei* microgametes, was used as negative control (Fig. 1E) (10).

### MG1 peptide binding inhibits fertilization

**Fig 2:**
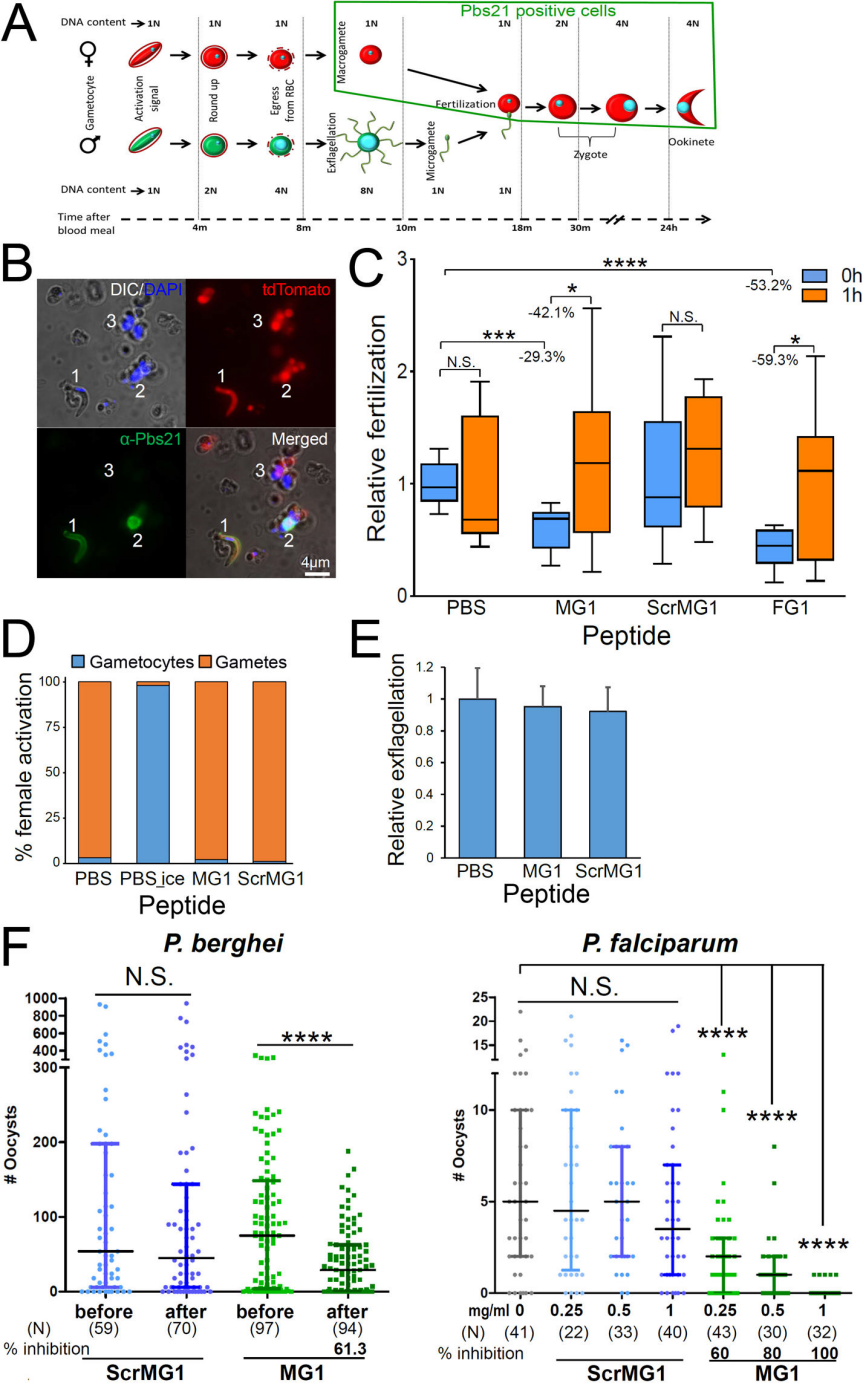
MG1 peptide binding inhibits fertilization. (**A**) Sexual development of the malaria parasite in the mosquito midgut. The green box indicates Pbs21 antibody-positive parasites shown in panels B and C modified from reference (14). m, minutes; h, hours. (**B**) Fluorescent staining of *P. berghei*-tdTomato 24 h after activation in ookinete medium identifies an ookinete (1), an unfertilized female gamete (2), and asexual stage parasites (3). (**C**) Gametogenesis was activated in ookinete medium with peptide addition (0.5 mg/mL) or not (PBS) at 0 h or 1 h. Parasites were fixed after 24 h and the fertilization rate determined as the ratio of ookinetes per total number of Pbs21 positive cells (female gametes plus ookinetes; total counts range from 914 to 1,155) in 60 microscopic fields under 400× magnification. The ratio of the no-peptide incubation (PBS) was set at 1. Data are pooled from three independent experiments, each with three technical replicates. Percent inhibition was calculated using median values (horizontal bar in the box). *P-*values were determined with Mann-Whitney U-test for PBS vs peptides and Wilcoxon test for 0 h vs 1 h (*, <0.05; ***, <0.001; ****, <0.0001). N.S., not significant. (**D**) The MG1 peptide does not interfere with female gametocyte activation, as determined by counting activated RFP-expressing *P. berghei* 820cl1m1cl1 female gametes that exited the host RBC (these do not co-localize with RBC membrane stained with anti-ter119 antibodies). The PBS-treated group served as a positive control with the same treatment, and similar samples incubated in ice (PBS_ice) served as a negative control. Data pooled from two independent experiments. MG1 and ScrMG1 peptide treatments have no significant difference with PBS control. (**E**) The MG1 peptide does not inhibit male exflagellation. Data are pooled from two independent experiments, each with four technical replicates. (**F**) MG1, but not scrambled MG1 (ScrMG1) peptide, inhibits *P. berghei* and *P. falciparum* oocyst formation. Inhibition assays were done by Passive Administration Feeding Assays (PAFA) for *P. berghei* and Standard Membrane Feeding Assays (SMFA) for *P. falciparum* (Fig. **S2**). For PAFA, oocyst numbers are given for mosquitoes that bit the infected mice before and after peptide injection. Data are pooled from three independent experiments. Vertical bars, range of the upper and the lower quartile; horizontal lines, medians; (N) ,number of mosquitoes analyzed. *P*-values were calculated with Mann-Whitney U-test (****, <0.0001); N.S., not significant.

We next sought to determine whether MG1 binding to male gametes interferes with fertilization. Following mosquito ingestion of infected blood, male and female gametocytes are quickly activated into gametes and this is followed rapidly by fertilization. The resulting zygote differentiates into a motile ookinete that 24 h later traverses the mosquito midgut epithelium. Female gametes, zygotes, and ookinetes can be stained by an anti-Pbs21 antibody (Fig. 2A) and ookinetes and unfertilized female gametes can be recognized by their shapes after antibody staining (Fig. 2B). For *in vitro* fertilization assays, *P. berghei*-tdTomato-infected (7) mouse blood was isolated and parasites were activated in ookinete medium in the presence or absence of synthetic peptides. After 24-h incubation, unfertilized parasites were stained with anti-Pbs21 antibodies (Fig. 2B) to determine fertilization rates (Fig. 2C). Compared to Phosphate buffered saline (PBS) control, MG1 and FG1 (positive control) peptides significantly inhibited fertilization rates as measured by decrease of ookinete numbers over total parasite numbers (including unfertilized female gametes). As a control, no significant differences in ookinete to total parasite numbers were observed when peptides were added at 1 h of incubation, after fertilization had occurred (Fig. 2A). Of note, the MG1 peptide did not inhibit female or male gametocyte activation (Fig. 2D and E). *In vitro* fertilization inhibition by MG1 peptide binding was confirmed by *in vivo* assays for rodent and human parasites (Fig. 2F). Our results show that the MG1 peptide selectively binds to male gametes (Fig. 1) and inhibits *P. berghei* and *P. falciparum* fertilization (Fig. 2C and F).

### An anti-MG1 peptide antibody binds to a female gamete ligand for interaction with male gametes

**Fig 3:**
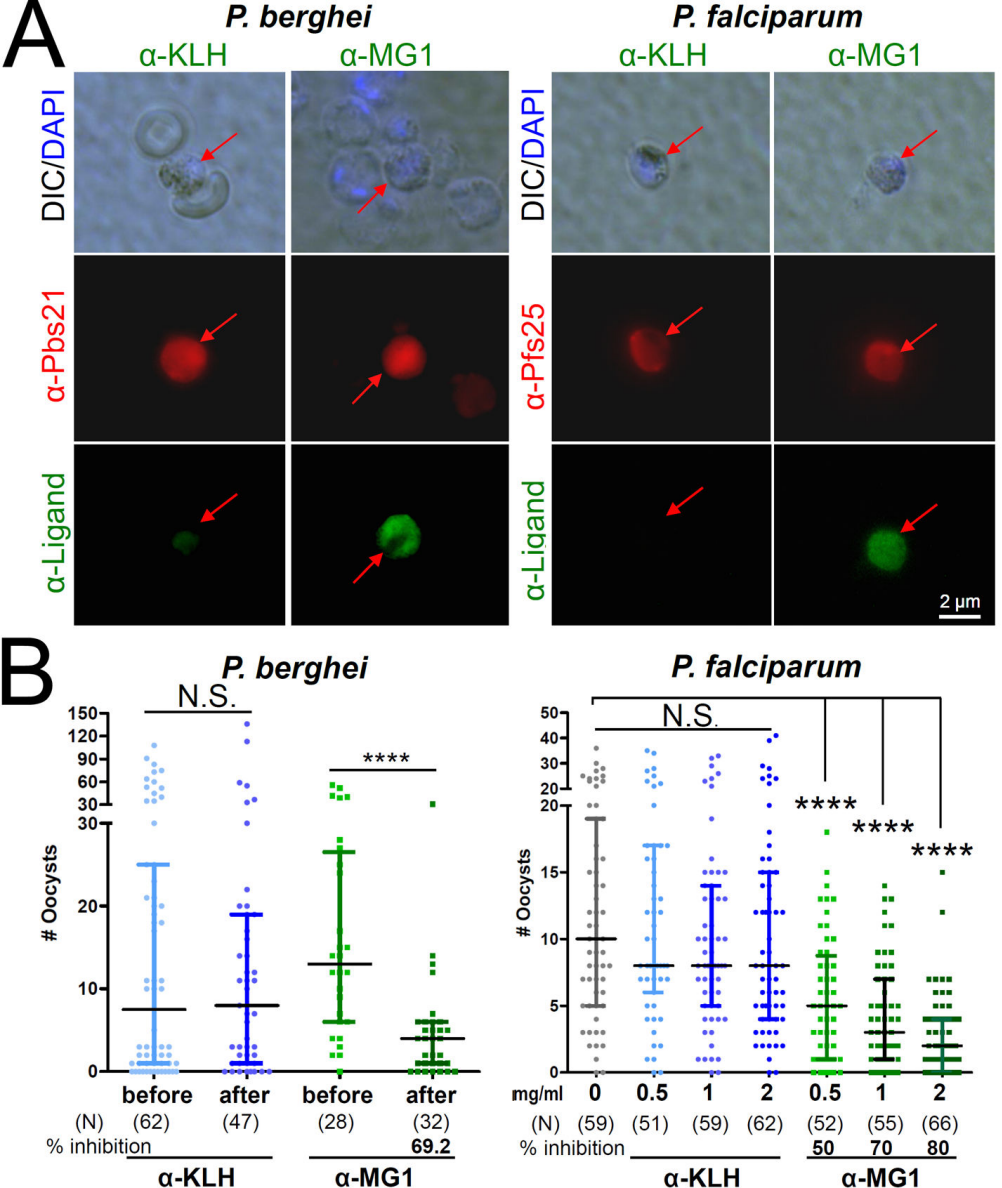
The MG1 peptide is a structural mimic of a *Plasmodium* female gamete ligand. (**A**) Anti-MG1antibodies recognize the surface of *P. berghei* and *P. falciparum* female gametes. Female gametes were labeled with an APC-conjugated anti-Pbs21 antibody for *P. berghei* and with an APC-conjugated anti-Pfs25 antibody for *P. falciparum*. Anti-KLH antibodies were used as a negative control. (**B**) Anti-MG1 immune serum, but not anti-KLH immune serum, inhibits fertilization. Inhibition assays were done by Passive Administration Feeding Assays (PAFAs) for *P. berghei* and Standard Membrane Feeding Assays (SMFAs) for *P. falciparum* (Fig. **S2**). For PAFA, oocyst numbers are given for mosquitoes that bit the infected mice before and after peptide injection. Data are pooled from three independent experiments. Vertical bars, range of the upper and the lower quartile, horizontal lines, medians; (N) ,number of mosquitoes analyzed. *P*-values (****, <0.0001). N.S., not significant. *P*-values were calculated with Mann-Whitney U-test.

We hypothesized that MG1 inhibits fertilization by binding to a male gamete receptor that interacts with a ligand on the female gamete surface (Fig. S2A). This hypothesis implies that MG1 and the relevant domain of the female gamete ligand protein share conformational properties. To test this hypothesis, we produced antibodies against MG1 fused to the Keyhole limpet haemocyanin (KLH) carrier protein. We found that anti-MG1 antibodies bind to both, *P. berghei* and *P. falciparum* female gametes, presumably by recognizing a ligand for fertilization (Fig. 3A). In agreement with this hypothesis, anti-MG1 antibodies, but not anti- KLH antibodies, inhibit *P. berghei* (69.2%) and *P. falciparum* (80%) oocyst development in mosquitoes (Fig. 3B).

### HSP90 on the female gamete surface is a ligand for male-gamete interaction

**Fig 4:**
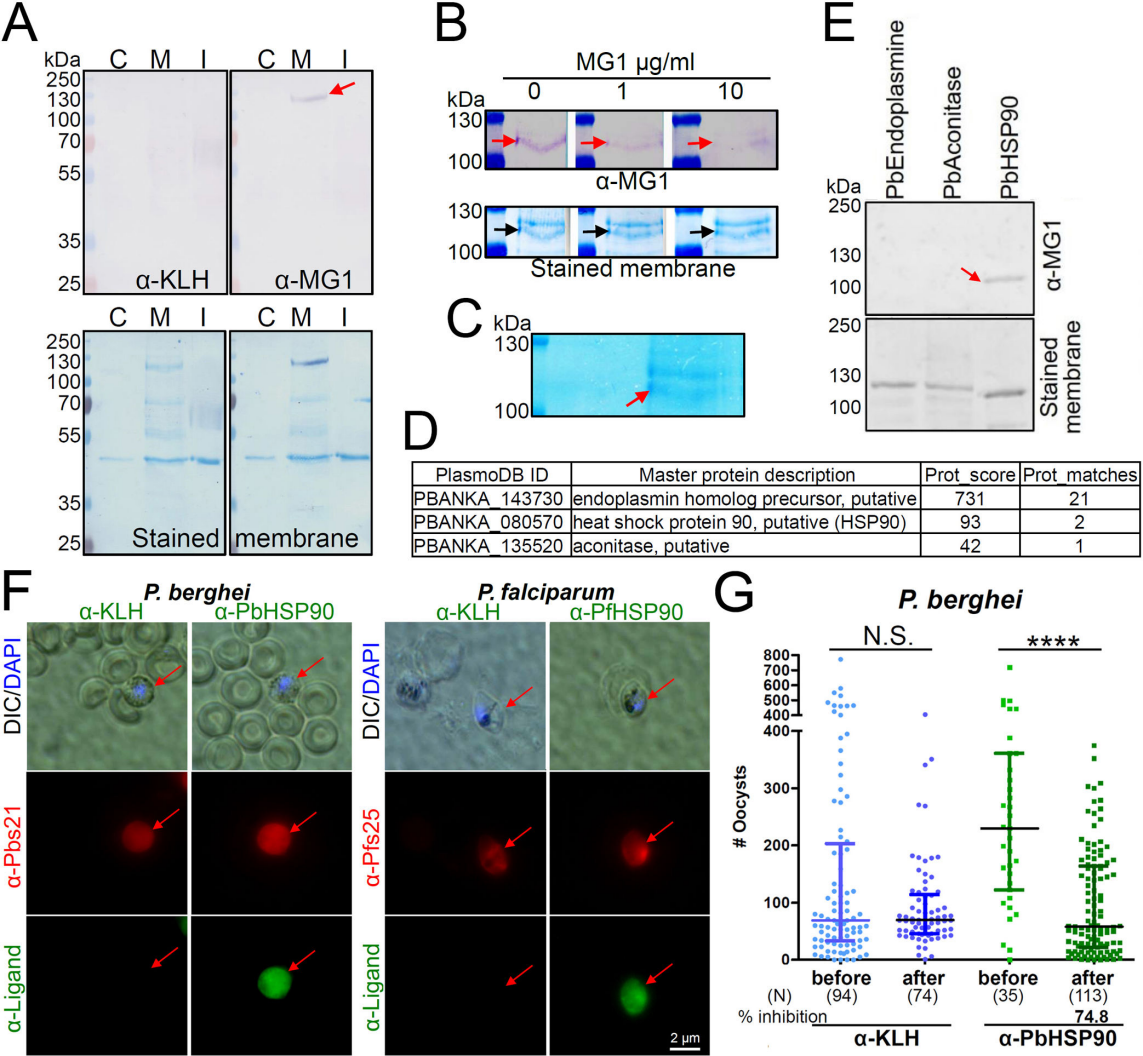
Anti-MG1 antibodies recognize HSP90 in the female gamete membrane fraction and inhibit fertilization. (**A**) Female gamete cytosolic (C), membrane (M), and insoluble (I) fractions were tested for anti-MG1 antibody binding. Anti-MG1 antibodies recognize a ~110 kDa membrane protein (red arrow). An anti-KLH antibody served as a negative control and the stained membranes served as loading references. (**B**) The MG1 peptide competitively inhibits the binding of anti-MG1 antibodies to the ~110 kDa protein in a dose-dependent manner (red arrows). Stained membranes served as a loading reference for the ~110 kDa band anti-MG1 target (black arrows). (**C and D**) Mass spectrometry analysis of the excised stained gel region corresponding to 110 kDa (C, red arrow) identified three female gamete membrane proteins (**D**). (**E**) The three candidate proteins identified by mass spectrometry were expressed as recombinant proteins. Only *Plasmodium* HSP90 is recognized by the anti-MG1 antibody. (**F**) Antibodies against *Plasmodium* HSP90 were tested for binding to the surface of non-permeabilized *P. berghei* and *P. falciparum* female gametes. Binding of anti-PbHSP90 and anti-PfHSP90 antibodies was detected with Alexa488-conjugated secondary antibody and female gametes were labeled with APC-conjugated anti-Pbs21 and anti-Pfs25 antibodies. Anti-KLH antibodies were used as a negative control. DIC, differential interference contrast microscopy; DAPI, nuclear stain (blue). (**G**) Anti-PbHSP90 antibodies, but not anti-KLH antibodies, inhibit *P. berghei* oocyst formation, as determined Passive Administration Feeding Assays (PAFAs) (Fig. S2B). For PAFA, oocyst numbers are given for mosquitoes that bite the infected mice before and after antibody injection. Data are pooled from two independent experiments. Vertical bars, range of the upper and the lower quartile; horizontal lines, medians; (N) ,number of mosquitoes analyzed. *P*-values (**** = <0.0001). N.S., not significant. *P*-value was calculated with Mann-Whitney U-test.

We sought to identify the surface female gamete molecule that is recognized by the anti-MG1 antibodies. Western blotting analysis revealed that anti-MG1 antibodies recognize a ~110 kDa protein in gamete membrane lysates that is not detected by the control anti-KLH antibodies (Fig. 4A). The binding of anti-MG1 antibodies to the ~110 kDa gamete protein was inhibited by the MG1 peptide in a dose-dependent manner, confirming specificity of the interaction (Fig. 4B). To determine the identity of the putative female gamete ligand, proteins in the ~110 kDa region were excised from a gel, followed by in-gel digestion, and analyzed by high-performance liquid chromatography mass spectrometry (Fig. 4C). Three parasite proteins, PBANKA_143730 (endoplasmin homolog precursor), PBANKA_080570 (heat shock protein 90, HSP90), and PBANKA_135520 (aconitase), were identified (Fig. 4D). The three recombinant proteins were produced, and Western blotting revealed that only recombinant PbHSP90 is recognized by the anti-MG1 antibody (Fig. 4E). Antibodies raised against recombinant *P. berghei* or *P. falciparum* HSP90 bind to the surface of female *P. berghei* and *P. falciparum* gametes (Fig. 4F). Importantly, anti-PbHSP90 antibodies strongly inhibit *P. berghei* oocyst formation in the mosquito (Fig. 4G). These results show that *Plasmodium* HSP90 acts as a female ligand for male gamete interaction.

## DISCUSSIONS

Using a phage display library, we identified the MG1 peptide that selectively binds to both activated *Plasmodium* male gametes and exflagellated microgametes. MG1 binding to these forms explains the peptide binding target molecule is expressed in the male gamete membrane surface during egression and remains on the microgamete for fertilization. MG1 peptide addition to cultures before induction of gamete activation and fertilization significantly decreased parasite development, whereas the inhibition was lost by adding the peptide 1 h after gamete activation and fertilization. This result supports the notion that MG1 actually acts as a fertilization inhibitor.

Our data support a model that MG1 and female gamete HSP90 share a structural domain(s) that interacts with a male gamete receptor required for fertilization. However, HSP90 is a multifunctional chaperon protein that interacts with diverse substrate and co-chaperon proteins involved in many cellular processes (16). Moreover, proteomics determined that *Plasmodium* HSP90 is present at most stages of the parasite life cycle, including sexual stages, merozoites, sporozoites, and on the surface of infected RBCs (17–19). Our results suggest that *Plasmodium* HSP90 on the female gamete surface has specific roles for fertilization. Of note, HSP90 has been identified as the most abundant mouse oocyte membrane protein and also has been found on the surface of human gametes (egg and sperm), and antibodies against this protein inhibit fertilization (20–22). Although known TBV candidate sex-specific gamete antigens, such as P48/45, P230, and P47, are involved in the processes of parasite development in the mosquito, targeting *Plasmodium* HSP90 has the potential for inhibiting multiple stages of the parasite life cycle. In particular, *Plasmodium* HSP90 has been identified as a drug target of blood-stage parasites (23) and now provides an important target for the transmission-blocking strategy for malaria control.

## MATERIALS AND METHODS

### *Plasmodium* parasites

*Plasmodium berghei* ANKA 2.34, *P. berghei*-tdTomato, *P. berghei* 820cl1m1cl1, and *P. berghei Δp48/45-Δp47* double-knockout lines (4) were propagated in 6- to 8-week-old female Swiss Webster mice (Envigo International Holdings). *Plasmodium falciparum* NF54 infectious gametocyte cultures were diluted to 0.03% gametocytemia before feeding to *Anopheles gambiae* mosquitoes, using an artificial membrane feeder. Gametocyte cultures and mosquitoes were provided by the Johns Hopkins Malaria Research Institute Parasite and Mosquito Core Facilities.

### *P. berghei* 820cl1m1cl1 gamete isolation and phage display library screening

Isolation of *P. berghei* male gametes was as described (14) with some modifications. Briefly, 3 days before infection, mice received intraperitoneal injection of 2 mg of phenylhydrazine (Sigma) in PBS to stimulate the production of reticulocytes. Mice were then infected with the *P. berghei* 820cl1m1cl1 blood-stage parasites (4) whose female gametocytes/gametes express RFP and male gametocytes/gametes express GFP. Two days after infection, mice received intraperitoneal injection of 0.2 mg pyrimethamine to eliminate asexual blood stage parasites. The gametocyte-enriched blood was activated in ookinete culture medium containing aphidicolin (0.5 nM) at room temperature. After 15 min, activated gametocytes were transferred to ice and purified using a magnetic column at 4°C. Purified gametes were kept on ice until sorting for GFP expression using a MoFlo Cell Sorter. A total of 5 × 10^5^ sorted male gametes were incubated with 1 × 10^11^ library phages in ookinete culture medium containing aphidicolin (0.5 nM) at room temperature for 30 min (Fig. S1A). The gametes were washed six times with ice-cold RPMI medium. Host *Escherichia coli* was added to the gametes to recover and amplify the bound phages. The amplified phages (1 × 10^11^ Colony forming units, CFUs) were used for the next round of selection. The process was repeated two more times for a total of three rounds of selection. After the third round, phage-infected *E. coli* were spread on tetracycline plates and the DNA sequence encoding the peptide was amplified by PCR from 39 individual colonies using the forward primer: 5′-CTTGTTCCTATGCTAAGCTTTG-3′ and the reverse primer: 5′-AGTAGCAGAAGCCTGAAGA-3′ for sequencing of the peptide-coding DNA.

### Gametocyte activation assay

Gamete egress from the RBC was used as a proxy for activation as previously described (14). RFP-expressing gametocytes were activated in ookinete culture medium for 15 min at room temperature in the presence or absence of 1 mg/mL of MG1 or ScrMG1 peptides. Egress was determined by quantifying the number of RFP-expressing *P. berghei* 820cl1m1cl1 female gametes not enclosed in RBC membrane, as detected with an Fluorescein isothiocyanate (FITC)-conjugated anti-Ter-119 antibody (Miltenyi Biotec). Male gamete exflagellation was measured by light microscopy to determine the effect of the peptides on male gametocyte activation. Aphidicolin-inhibited exflagellation did not affect egress of the male gamete from the RBC detected by labeling the RBC membrane-associated cytoskeleton with an anti-spectrin antibody (Sigma) (Fig. 1B).

### Immunofluorescence assay

For immunofluorescence assays, non-permeabilized *P. berghei* and *P. falciparum* gametocytes were fixed in 4% paraformaldehyde for 1 h and blocked with 4% Bovine serum albumin (BSA) for 1 h. Then, gametocytes were incubated with 10 µg/mL peptide or 0.5% antiserum in PBS. Peptide or antibody binding was visualized with fluorescent streptavidin or secondary antibody, respectively. Fluorescent *P. berghei* 820cl1m1cl1 gametes were permeabilized in 0.1% Triton X100 in PBS for 15 min after peptide and streptavidin binding, for removal of cytosolic RFP or GFP protein.

### In-gel digestion and proteomics analysis

For gel band excision, clean and sterile razors were used to cut the desired band/slice in a process-protection hood. The slice was further cut into 1 × 1 mm pieces prior to de-staining, reduction and alkylation, tryptic digestion and peptide extraction. The extracted peptides were lyophilized by speed-vac and resuspended in 2% acetonitrile, 97.9% water, and 0.1% formic acid buffer for Liquid Chromatography with tandem mass spectrometry (LC-MS/MS) analysis and database searching for the protein identification.

### Recombinant protein expression and Western blotting

*Plasmodium berghei* proteins (endoplasmin, aconitase, and PbHSP90 in Fig. 4D and E) and *P. falciparum*-specific HSP90 fragment (comprising amino acids 221–309, which are missing in PbHSP90) were expressed using pBad202 Directional TOPO Expression Kit (Invitrogen). Parasites and recombinant proteins were harvested, pelleted, resuspended with 1× SDS gel loading buffer, and boiled for 10 min. Protein lysates were fractionated by 10% SDS-PAGE and then transferred onto a polyvinylidene difluoride (PVDF) membrane. Membranes were incubated for 1 h in blocking buffer (0.1% Tween 20 and 4% milk in PBS), followed by addition of a primary antibody. Antibody binding was visualized with alkaline phosphatase–conjugated secondary antibody (Promega). PVDF membranes were stained with Coomassie dye as loading reference. For Western blotting assay in Fig. 4A and B, sorted female gametocytes were used for sub-cellular fractionation as described previously (13). Briefly, female gametocytes were harvested with buffer 1 (0.01% digitonin, 10 mM piperazine-N,N′-bis(2-ethanesulfonic acid) (PIPES), pH 6.8, 300 mM sucrose, 100 mM NaCl, 3 mM MgCl_2_, and 5 mM EDTA) and rotated for 10 min at 4°C. After 1-min centrifugation at 16,900 × *g*, the supernatant (cytosolic fraction) was collected and the cell pellet was washed with buffer 1. The pellet was then resuspended with buffer 2 (0.5% Triton X-100, 10 mM PIPES, pH 7.4, 300 mM sucrose, 100 mM NaCl, 3 mM MgCl_2_, and 3 mM EDTA) and rotated for 20 min at 4°C. After a 1-min centrifugation (16,900 *g*), the supernatant (membrane fraction) was collected and the cell pellet was washed with buffer 2. The pellet was then resuspended in 6.5 M urea (insoluble fraction).

### Antibody generation and inhibition assays

To generate antibodies, 6- to 8-week-old female Swiss Webster mice were immunized with 50 µg antigen per mouse (KLH conjugated-MG1 peptide, KLH alone, recombinant PbHSP90 full-length protein, PfHSP90 fragment protein) using 50% AddaVax adjuvant (InvivoGen). Priming subcutaneous injection was followed by triple boosts at 2-week intervals. At 10 days after the last boost, immunized mice were bled for immune sera collection.

For Passive Administration Feeding Assays (Fig. S2B), 100 µL of antisera per mouse was injected through tail vein, and for Standard Membrane Feeding Assays, doses of antibody were denoted on the Figures. Expression of full-length PfHSP90 was not successful. Since the anti-PfHSP90 antibody was generated with only a fragment of PfHSP90, no inhibition assays were performed.

### Statistical analyses

The two tailed Mann-Whitney U-test was used for non-parametric analysis. The Wilcoxon test was used for non-parametric analysis of two paired groups in Fig. 2C.

## Data Availability

The data underlying Fig. 1 to 4 are in the published article.

## SUPPLEMENTAL MATERIAL

Supplemental figures mbio.03142-23-s0001.docx Figure S1 to S3.

ASM does not own the copyrights to Supplemental Material that may be linked to, or accessed through, an article. The authors have granted ASM a non-exclusive, world-wide license to publish the Supplemental Material files. Please contact the corresponding author directly for reuse.
