# Supplementary material for: Plasmodium female gamete surface HSP90 is a key determinant for fertilization
Source: mBio. 2023 Dec 22;15(2):e03142-23. doi: 10.1128/mbio.03142-23 (PMC10865824; doi:10.1128/mbio.03142-23)
Supplement: Supplemental figures — Figure S1 to S3. [file mbio.03142-23-s0001.docx]

**Supplementary Materials**

**
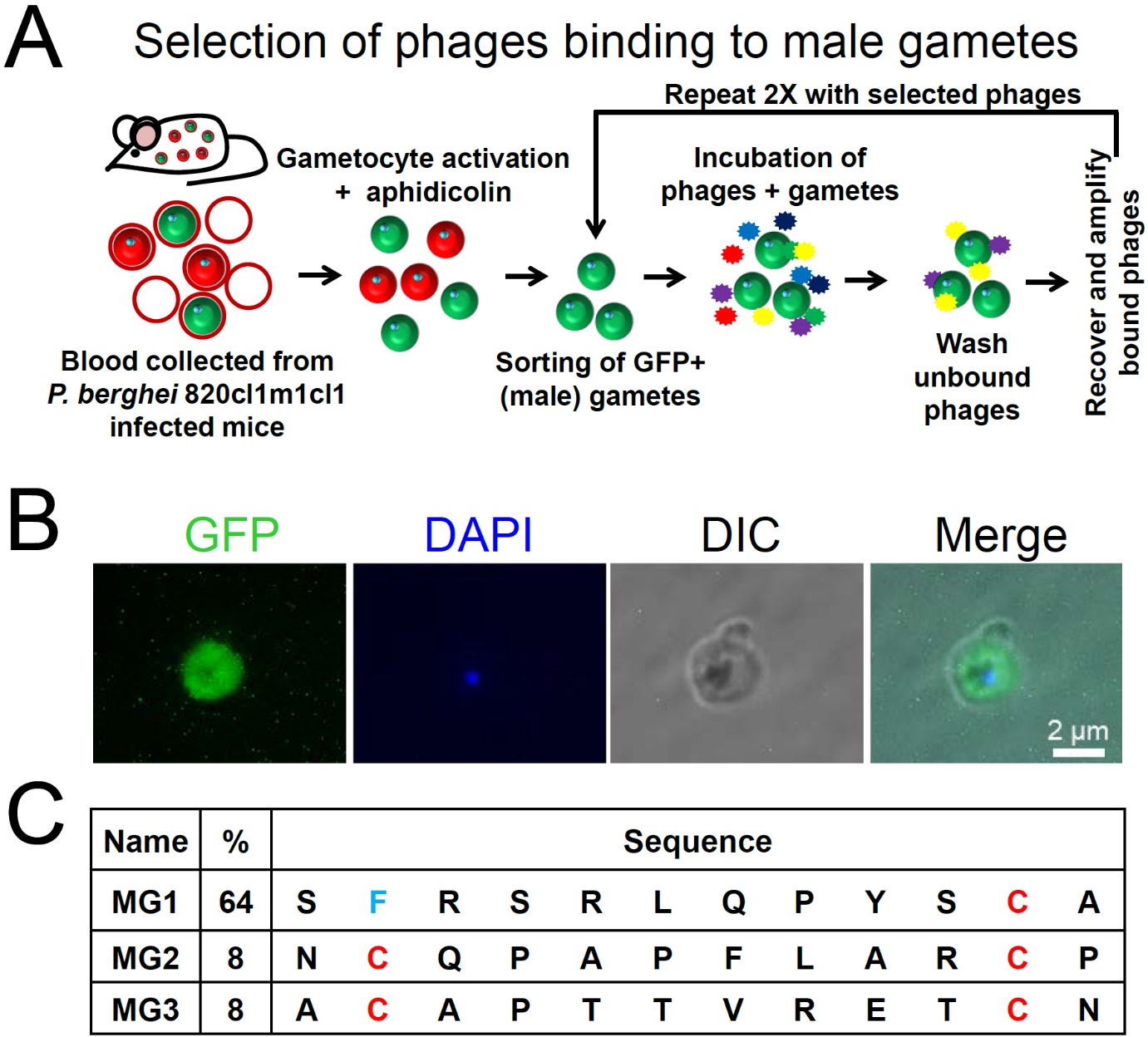
 Fig. S1. Phage display library screening to identify peptides with high-binding affinity to *Plasmodium* male gametes.** (A) Schematic diagram of the phage peptide-display screening targeting *Plasmodium* fertilization (modified from *14*). *Plasmodium berghei* 820cl1m1cl1 parasites yield green-fluorescent male gametocytes, red-fluorescent female gametocytes and non-fluorescent asexual parasites (*4*). Gametocytes were activated in the presence of aphidicolin, a DNA polymerase inhibitor, to block male gamete exflagellation and avoid fertilization. Pure GFP-expressing non-exflagellated male gametes obtained by fluorescence activated cell sorting (FACS) were incubated with a phage display library that has a complexity of 1.5 X 10^9^ different peptides. Phages that bound to male gametes were recovered and amplified. The selection process was repeated two more times. (B) Image of an activated GFP-expressing male gamete isolated by FACS and used for screening. (C) Amino acid sequences of 39 male-gamete-binding peptides displayed by phages after the third round of selection. The percentage of phages displaying each peptide sequence is indicated. Peptides sequences with single occurrence are not shown. The library was constructed with random amino acids at all positions, except for the cysteines at positions 2 and 11, which make a disulfide bond, generating an eight-amino acid loop. The MG1 peptide is exceptional in that the cysteine in position 2 mutated to a phenylalanine (TGT to TTC)

**
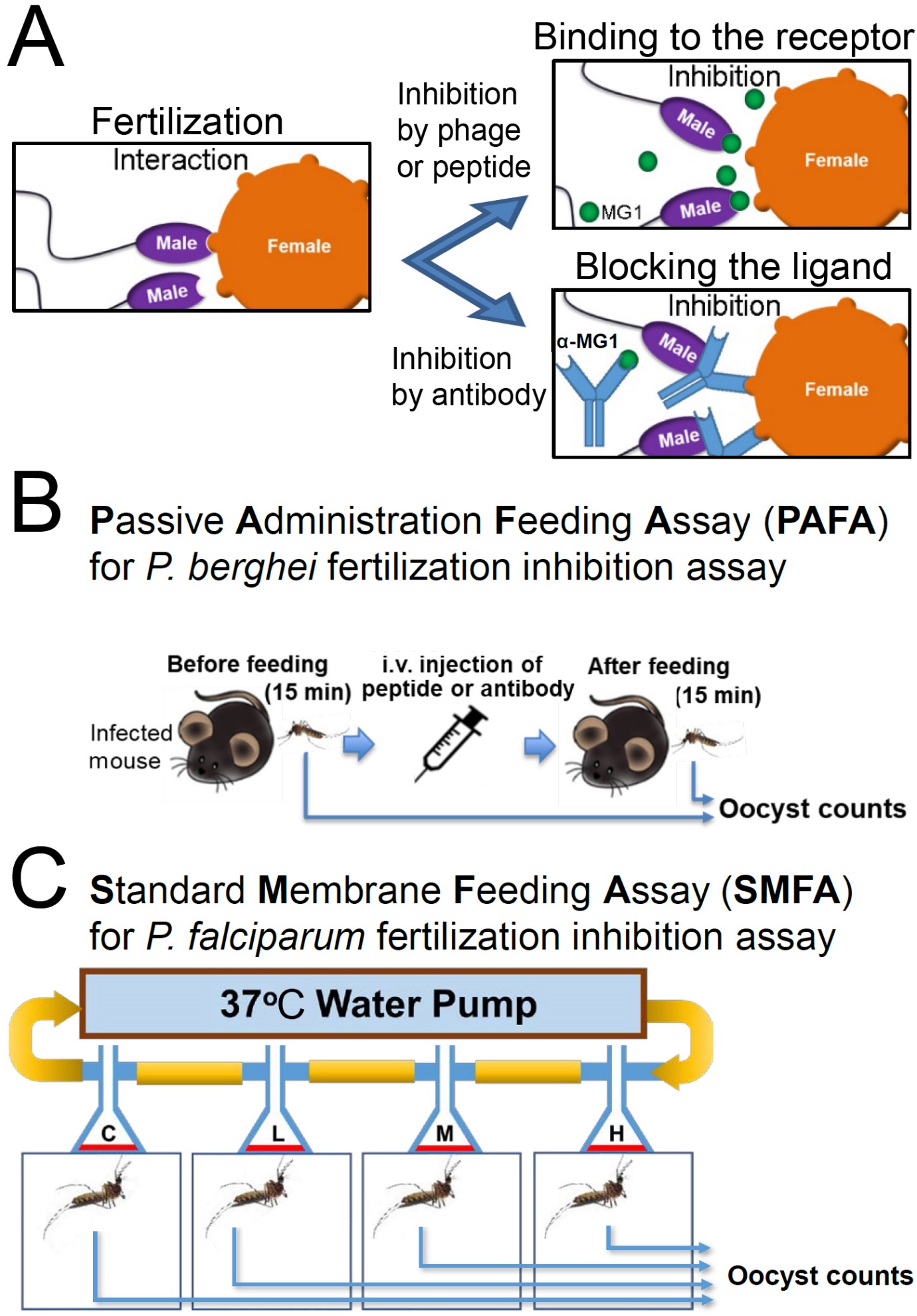
Fig. S2. Mosquito transmission-blocking assays.** (A) Left panel: we hypothesize that fertilization requires the interaction between a female ligand and a male receptor. The panels on the right illustrate two fertilization inhibition assays to test this working hypothesis. Upper panel: the MG1 peptide binds to the male gamete receptor and inhibits fertilization; Lower panel: the MG1 peptide mimics the structure of a female surface ligand that interacts with a male receptor, hence anti-MG1 antibodies block fertilization by binding to the female ligand. (B) The **P**assive **A**dministration **F**eeding **A**ssays (PAFA) was used to test the hypotheses illustrated in (A). A group of *An. gambiae* mosquitoes fed for 15 min on a *P. berghei*-infected mouse before injection of the transmission-blocking agent. Next, the mouse was injected intravenously with 10^11^ CFU phages, or 600 µg peptide or 200 µl immune sera. After 15 min of recovery to allow injected agent distribution in the mouse blood stream, a second group of mosquitoes were fed on the same mouse. Therefore, control (before injection) and experimental (after injection) mosquitoes ingest approximately the same number of parasites and oocyst numbers can be compared. Mosquito midguts are dissected 10 d post-infection to determine the number of oocysts per midgut. (C) For *P. falciparum*, we used the **S**tandard **M**embrane **F**eeding **A**ssay (SMFA) as follows. Membrane feeders were prepared with same amount of *P. falciparum* gametocyte culture containing different concentrations (C, no agent control; L, low concentration; M, medium concentration; H, high concentration) of transmission-blocking agents. A group of *An. gambiae* mosquitoes were fed on each feeder for 30 min. Mosquito midguts were dissected 7 d post-infection to determine the number of oocysts per midgut.

**
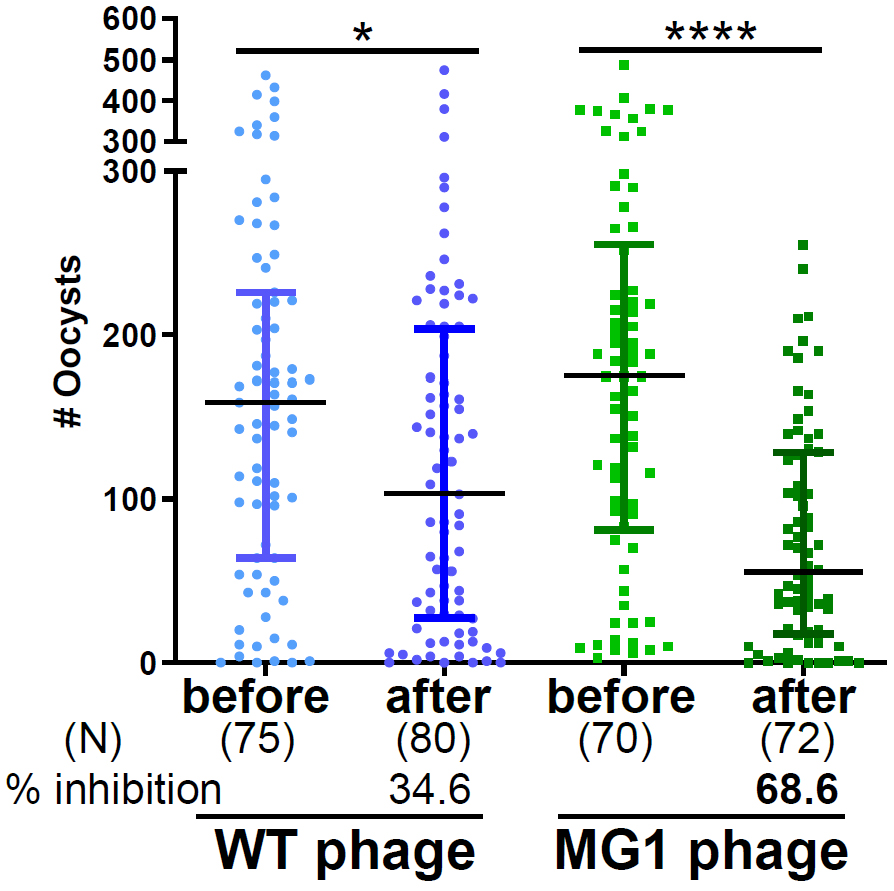
**

**Fig. S3. The MG1 phage inhibits oocyst formation.** PAFA assays (Fig. S2) were done with the MG1 phage and the wild type phage as control. Data pooled from two independent experiments. The vertical bars in the scattered plots show the range of the upper and the lower quartile and the horizontal lines the medians. The number in parenthesis denotes number of mosquitoes (N) analyzed. *P*-values (*, <0.05; ****, <0.0001) were calculated with the Mann-Whitney U test.
